# Supplementary material for: Self-reported attitudes, knowledge and skills of using evidence-based medicine in daily health care practice: A national survey among students of medicine and health sciences in Hungary
Source: PLoS One. 2019 Dec 27;14(12):e0225641. doi: 10.1371/journal.pone.0225641 (PMC6934312; doi:10.1371/journal.pone.0225641)
Supplement: S3 Questionnaire — (PDF) [file pone.0225641.s003.pdf]

A bizonyítékokon alapuló orvoslás gondolatkörének a megjelenése az orvosi- és egészségtudományi képzésben

KÉRDŐÍV ORVOSTANHALLGATÓK SZÁMÁRA

<u>Háttér információ</u>

1. Hallgató neme
   a. férfi
   b. nő

2. Hányadik évfolyamon hallgatója vagy?
   a. első
   b. második
   c. harmadik
   d. negyedik
   e. ötödik
   f. hatodik

3. Dolgoztál már korábban az egészségügyben (legalább 1 évet)?
   a. igen
   b. nem

4. Tanulmányaid mellett részt veszel kutatócsoport munkájában (pl. TDK munka keretében)?
   a. igen
   b. nem

5. A közeli hozzátartozóid között (szülők, testvérek, házastárs) van olyan, aki az egészségügyben dolgozik?
   a. igen
   b. nem

6. Ha igen, hatással volt ez a pályaválasztásodra?
   a. igen
   b. nem

7. Milyen gyakran olvasol szakmai folyóiratokat?

   a. naponta
   b. hetente
   c. havonta vagy ritkábban
   d. soha

8. Rendelkezel saját számítógéppel?
    a. igen
    b. nem

9. Van internet hozzáférésed?
    a. igen
    b. nem

10. Van ingyenes internet hozzáférésed?
    a. igen
    b. nem

11. A következő kereső felületek közül melyiket használtad már orvosi/ egészségügyi információ szerzésére?
    a. Google
    b. Google scholar
    c. Wikipedia
    d. Pubmed/Medline
    e. Medscape
    f. Cochrane Library

12. Mit tekintesz az egészségügyi információszerzés fő forrásának?
    a. nyomtatott szakkönyvek
    b. nyomtatott folyóiratok
    c. elektronikus szakkönyvek
    d. elektronikus folyóiratok
    e. elektonikus média
    f. szakmai irányelvek
    g. szórólapok
    h. oktatási jegyzetek
    i. egészségügyi szakemberek véleményének megismerése

13. Vettél már részt szakmai képzésen (kurzus, tanfolyam), ahol a bizonyítékokon alapuló orvoslással (BAO, EBM) kapcsolatos ismereteket szereztél?
    a. igen
    b. nem

14. Ha igen, hasznosnak tartottad a képzést a további tanulmányaid illetve a későbbi munkád szempontjából?
    a. igen
    b. nem

15. Ha nem, hasznosnak tartanál egy ilyen képzést a további tanulmányaid illetve a későbbi munkád szempontjából?

a. igen
b. nem

16. Ha igen, melyik évfolyamon lenne szerinted hasznos az EBM oktatása?
    a. az 1.-2. évfolyamon
    b. a 3.-4. évfolyamon
    c. az 5.-6. évfolyamon

17. Szerepet játszik-e az adott egészségügyi információ nyelve abban, hogy elolvasod-e?
    a. Igen, csak magyarul szeretek olvasni
    b. Igen, csak angolul szeretek olvasni
    c. Nem, az angol és magyar nyelvű forrásokat egyaránt elolvasom
    d. Nem, sőt, olvastam már olyan forrást is, ami egyéb (nem angol vagy magyar) nyelven íródott

<u>EBM tudás felmérés</u>:

Milyennek ítéled a készségeidet a következő területeken?

	Gyenge	Van valamennyi tapasztalatom	Átlagos	Átlagosnál jobb	Haladó szintű
	(1)	(2)	(3)	(4)	(5)
Szakmai irodalom keresése					
Keresés online adatbázisokban					
Klinikai kutatásokat leíró közlemények kritikus értékelésé					
Olyan fontos klinikai területek azonosítása, ahol még nem áll rendelkezésre elég szakirodalom					
A rendelkezésre álló szakirodalom kritikus megítélése					
Betegek szempontjából fontos kérdések azonosítása					

<u>Önbevalláson alapuló fogalomismeret</u>: **Mennyire ismered az alábbi fogalmakat?**

	**Értem a jelentését, és másoknak is el tudnám magyarázni**	**Többé-kevésbé értem a jelentését**	**Nem ismerem a jelentését, de szeretném megismerni**	**Nem ismerem a jelentését, de szerintem nem is hasznos számomra**	**Nincs véleményem ezzel kapcsolatosan**
bizonyítékokon alapuló orvoslás (evidence-based medicine)					
eredeti kezelési szándék szerinti elemzés (intention-to-treat analysis)					
esetszám (sample size)					
esettanulmány (case study)					
kohorsz vizsgálat (cohort study)					
konfidencia intervallum					
kontrollált klinikai vizsgálat					
lemorzsolódás (lost to follow-up)					
meta-analízis					
NNT (number needed to treat)					
randomizálás (randomization)					
szakmai irányelv (guideline)					
szisztematikus irodalmi áttekintés (systematic review)					

Attitűd felmérés: Mennyire értesz egyet a következő állításokkal?

	Egyáltalán nem értek vele egyet	Nem értek egyet	Semleges	Egyet értek	Kifejezetten egyet értek
A bizonyítékokon alapuló orvoslás (BAO) fontos a gyakorló orvos munkája szempontjából					
Szeretném, hogy a tanulmányaim során azon képességeim fejlődjenek, mely a bizonyítékokon alapuló orvoslás (BAO) gyakorlati orvosi munka során történő alkalmazásához szükséges					
A bizonyítékokon alapuló orvoslás fontos a betegek optimális ellátásához					
A bizonyítékokon alapuló orvoslás elősegíti a betegek ellátásával kapcsolatos döntéshozatalt					
A bizonyítékokon alapuló orvoslás figyelembe veszi a gyakorló orvosok egyéni tapasztalatait					
A bizonyítékokon alapuló orvoslás figyelembe veszi a betegek kezeléssel kapcsolatos véleményét, preferenciáit					
Fontos, hogy a kutatási eredmények beépüljenek az orvosi gyakorlatba					
Minden klinikai vizsgálat azonos értékű					
A bizonyítékokon alapuló orvoslás gyakorlati alkalmazása megvalósíthatatlan terhet ró az egészségügyben dolgozókra					
A betegek ellátásával kapcsolatban felmerülő					

kérdéseimre a legmegfelelőbb választ szakkönyvekből kaphatom					
Leendő egészségügyi dolgozóként fontosnak tartom az egész életen át tartó tanulást					
