# Supplementary material for: Self-reported attitudes, knowledge and skills of using evidence-based medicine in daily health care practice: A national survey among students of medicine and health sciences in Hungary
Source: PLoS One. 2019 Dec 27;14(12):e0225641. doi: 10.1371/journal.pone.0225641 (PMC6934312; doi:10.1371/journal.pone.0225641)
Supplement: S4 Questionnaire — (PDF) [file pone.0225641.s004.pdf]

Evidence-based medicine in medical and health sciences education

QUESTIONNAIRE FOR MEDICAL STUDENTS (English translation)

Background information

1. Gender
   a. male
   b. female

2. Which university year are you currently in?
   a. first year
   b. second year
   c. third year
   d. fourth year
   e. fifth year
   f. sixth year

3. Have you already worked in healthcare (at least 1 year)?
   a. Yes
   b. No

4. Do you participate in the activity of a research group alongside your studies (for example as member of the Scientific Students' Association)?
   a. Yes
   b. No

5. Do you have any close family members (parents, siblings, spouse) working in health care?
   a. Yes
   b. No

6. If YES to question Nr 5: Did it affect your career choice?
   a. Yes
   b. No

7. How often do you read scientific literature?
   a. daily
   b. weekly
   c. monthly or less frequently
   d. never

8. Do you have an own computer?
    a. Yes
    b. No

9. Do you have internet access?
    a. Yes
    b. No

10. Do you have free internet access?
    a. Yes
    b. No

11. Which of the following search engines have you already used to obtain medical / health information?
    a. Google
    b. Google scholar
    c. Wikipedia
    d. Pubmed/Medline
    e. Medscape
    f. Cochrane Library

12. What do you see as the main source of health information?
    a. printed books
    b. printed journals
    c. online books
    d. online journals
    e. electronic media
    f. professional guidelines
    g. leaflets
    h. lecture notes
    i. opinion of health professionals

13. Have you already participated in a professional training course where you acquired evidence-based medicine (EBM)-related knowledge?
    a. Yes
    b. No

14. If YES to question Nr. 13: Did you find the training useful for your further studies and for your future work?
    a. Yes
    b. No

15. If NO to question Nr. 13: Would you consider such a training useful for your further studies and for your future work?
    a. Yes
    b. No

16. If YES to question Nr. 15: In which university year would you consider EBM education useful?
    a. in the 1st - 2nd year
    b. in the 3rd - 4th year
    c. in the 5th – 6th year

17. Does the language of health information play a role in making sure you read it?
    a. Yes, I only like to read in Hungarian
    b. Yes, I only like to read in English
    c. No, I read both English and Hungarian information resources
    d. No, indeed, I have already read information sources written in a language other than English or Hungarian

<u>EBM-related skills</u>:

How would you rate your skills in the following areas?

	**Poor (1)**	**Limited experience (2)**	**Average (3)**	**Above average (4)**	**Advanced (5)**
Locating professional literature					
Searching online databases					
Critical appraisal of a scientific publication describing findings from clinical research					
Identifying fields where not enough scientific literature is available to answer a specific clinical question					
Critical appraisal of available scientific literature					
Identifying patient-relevant clinical questions					

<u>Knowledge of EBM-related terms</u>: How familiar are you with the following concepts?

	I understand and I could explain to others	Some understanding	I do not understand, but would like to understand	I do not understand, but I think, it wouldn't be helpful to me to understand	No idea about this
evidence-based medicine					
intention-to-treat analysis					
sample size					
case study					
cohort study					
confidence interval					
controlled clinical trial					
lost to follow-up					
meta-analysis					
NNT (number needed to treat)					
randomization					
professional guideline					
systematic literature review					

<u>Attitude Survey</u>: How much do you agree with the following statements?

	Strongly disagree	Disagree	Neutral	Agree	Strongly agree
Evidence-based medicine is important for the medical practitioner's work					
During my studies, I would like to develop the skills needed to apply evidence-based medicine in practical medical work					
Evidence-based medicine is important for optimal patient care					
Evidence-based medicine facilitates patient decision-making					
Evidence-based medicine takes into account the individual professional experience of physicians					
Evidence-based medicine takes into account the views and preferences of patients regarding their treatment					
It is important that research results are integrated into medical practice					
All clinical trials are of equal value					
The practical application of evidence-based medicine puts an unrealistic burden on health professionals in daily routine patient care					
Medical books give me the best answer to my questions regarding patient care					
As a future health worker, I consider lifelong learning important					
