# Supplementary material for: Self-reported attitudes, knowledge and skills of using evidence-based medicine in daily health care practice: A national survey among students of medicine and health sciences in Hungary
Source: PLoS One. 2019 Dec 27;14(12):e0225641. doi: 10.1371/journal.pone.0225641 (PMC6934312; doi:10.1371/journal.pone.0225641)
Supplement: Attachment — Submitted filename: Review.docx [file pone.0225641.s005.docx]

Many thanks for the opportunity to review this manuscript, which I enjoyed reading. A report from an online survey relating to attitudes and perceptions of evidence-based medicine in presented.

Evidence based medicine (EBM) is important in all aspects of healthcare, so research which considers students’ learning attitudes is clearly very valuable. The inclusion of both medical students and other healthcare students in this study is a strength.

 Please consider the following comments.

1. The methods are generally well reported, however there are a few omissions which would be useful to clarify: What language was the survey conducted in?; Did any exclusion criteria apply (i.e. students with formal research backgrounds)?; It would be useful to provide a copy of the questionnaire, or at least a full outline of the questions in supplementary format.

2. National completion of the survey is a strength since it helps to increase generalisability. To what extent might these results be useful beyond Hungary? - please consider commenting on this in the discussion.

3. I agree with the study limitations, but these need further elaboration. The key limitation seems to be the role of response bias. I think a fuller discussion about this is required i.e. to what extent is a sample of 17% generalisable to the Hungarian healthcares student population; what is the likely impact of self-responses to survey questions (bias), etc.

4. There is clearly a large appetite for students learning the principles of EBM. This study identifies some key attitudes and perceptions, but how might these results be taken forward? I would suggest that this is briefly considered in the Discussion.

5. A discretionary comment: Often institutions do not look favourably on centre-specific comparisons (i.e. comparisons of data between Universities) unless permission is sought. This may be different in Hungary. To avoid problems later, I would suggest that the authors check to ensure this is permitted.

6. The manuscript is generally written well, the tables are well presented, and the references seem appropriate.
